# Prognostic and Immunological Role of PPP1R14A as a Pan-Cancer Analysis Candidate

**DOI:** 10.3389/fgene.2022.842975

**Published:** 2022-05-17

**Authors:** Zhaotao Wang, Rihong Huang, Haojian Wang, Yuecheng Peng, Yongyang Fan, Zejia Feng, Zhaorong Zeng, Yunxiang Ji, Yezhong Wang, Jiajie Lu

**Affiliations:** ^1^ Department of Neurosurgery, Institute of Neuroscience, The Second Affiliated Hospital of Guangzhou Medical University, Guangzhou, China; ^2^ Department of Clinical Medicine, The Second Clinical School of Guangzhou Medical University, Guangzhou, China

**Keywords:** PPP1R14A, pan-cancer, prognosis, diagnosis, genetic alternation, immune, phosphorylation

## Abstract

Despite emerging evidence revealing the remarkable roles of protein phosphatase 1 regulatory inhibitor subunit 14A (PPP1R14A) in cancer tumorigenesis and progression, no pan-cancer analysis is available. A comprehensive investigation of the potential carcinogenic mechanism of PPP1R14A across 33 tumors using bioinformatic techniques is reported for the first time. PPP1R14A is downregulated in major malignancies, and there is a significant correlation between the PPP1R14A expression and the prognosis of patients. The high expression of PPP1R14A in most cases was associated with poor overall survival (OS), disease-specific survival (DSS), and progress-free interval (PFI) across patients with various malignant tumors, including adrenocortical carcinoma (ACC) and bladder urothelial carcinoma (BLCA), indicated through pan-cancer survival analysis. Receiver operating characteristic (ROC) analysis subsequently exhibited that the molecule has high reference significance in diagnosing a variety of cancers. The frequency of PPP1R14A genetic changes including genetic mutations and copy number alterations (CNAs) in uterine carcinosarcoma reached 16.07%, and these alterations brought misfortune to the survival and prognosis of cancer patients. In addition, methylation within the promoter region of PPP1R14A DNA was enhanced in a majority of cancers. Downregulated phosphorylation levels of phosphorylation sites including S26, T38, and others in most cases took place in several tumors, such as breast cancer and colon cancer. PPP1R14A remarkably correlated with the levels of infiltrating cells and immune checkpoint genes. Our research on the carcinogenic effect of PPP1R14A in different tumors is comprehensively summarized and analyzed and provides a theoretical basis for future therapeutic and immunotherapy strategies.

## Introduction

Cancer is a persistent public health challenge of concern prevalent globally. According to estimates from 2019 Global Cancer Statistics, cancer is a major obstacle to the life expectancy growth in the 21st century among the first or second leading cause of death before the age of 70 years in 112 of 183 countries, and it ranks third or fourth in further 23 countries (Sung et al., 2021). Progress with early diagnosis and innovative methods for reducing mortality have made great efforts in various cancers. However, the incident rate of malignant cancer is increasing. There was a projection that there will be approximately 1.2 million new cancer cases and 400,000 cancer deaths in 2020 as compared with 2018, which is a major concern for public human health (Ferlay et al., 2015; Gao et al., 2017; Bray et al., 2018; Author Anonymous, 2020). Due to the complexity of tumorigenesis, a pan-cancer analysis of the same gene has attracted increased attention, which has contributed to identify common phenotypic characteristics and deeply understand the internal regulation mechanism of key molecules and the relationship between potential clinical prognosis (Kang et al., 2021).

Protein phosphatase 1 regulatory inhibitor subunit 14A (PPP1R14A), belonging to the protein phosphatase 1 (PP1) inhibitor family, is often referred to by the alias 17 kDa PKC-potentiated inhibitory protein of PP1 (CPI-17) and has more than 1,000 times inhibitory activity during phosphorylation, which results in a molecular switch to regulate the phosphorylation state of PPP1CA substrate and smooth muscle contraction. Previous studies have shown that PPP1R14A is associated with a pivotal role in the occurrence and development of tumors, including sporadic vestibular glioma, human melanoma, and schwannoma (Hagel et al., 2016; Riecken et al., 2016; Xu et al., 2020). In addition, Jin et al. demonstrated that the downregulation of CPI-17 induces merlin dephosphorylation, thereby inhibiting Ras activation and retarding the tumorigenic transformation phenotype (Jin et al., 2006). However, most studies on the function of PPP1R14A in cancers were restricted to specific cancer types. Therefore, deeply examining the regulatory functions and molecular mechanisms of PPP1R14A in pan-cancer is vital to gain new insights into relevant carcinogenic mechanisms and inform strategies and direction for the clinical treatment of cancers.

In this study, we performed a pan-cancer expression analysis of PPP1R14A based on the TCGA database, identified a key molecule through a series of experiments including exploring PPP1R14A differential expressions, clinical survival prognosis, genetic alteration, promoter DNA methylation, protein phosphorylation, immune infiltration landscape, and putative signaling pathway, and explored the underlying mechanism in tumorigenesis and tumor suppression across different cancer species. The analysis of PPP1R14A provides new insights into the carcinogenic role of PPP1R14A across multiple malignancies and provides further understanding of the individual management for cancer precision therapy.

## Materials and Methods

### Expression Patterns, Phosphorylation, and Promoter Methylation of Protein Phosphatase 1 Regulatory Inhibitor Subunit 14A in Human Pan-Cancer

To compare PPP1R14A’s expression level between tumor and matched normal tissues across all TCGA cancer types, the “Gene_DE” module in “Exploration” of the TIMER2.0 database, providing comprehensive resource for systematic analysis of immune infiltrates across diverse cancer types based on TCGA cohorts (Li et al., 2020), was applied in this analysis, and the dysregulation of PPP1R14A expression between various types of cancer and normal tissues was investigated by combining the data, which had been uniformly processed by the Toil process in UCSC Xena (https://xenabrowser.net/datapages/) (Vivian et al., 2017; Goldman et al., 2020), for normal tissues from the GTEx and The Cancer Genome Atlas (TCGA) databases and visualized by using the R ggplot2 (version: 3.3.3) package.

The UALCAN portal (http://ualcan.path.uab.edu/analysis-prot.html) (Chandrashekar et al., 2017), an interactive web resource for analyzing cancer OMICS data, allows for conducting protein expression analysis of Clinical Proteomic Tumor Analysis Consortium (CPTAC) datasets. The expression levels were explored, of the total protein or phosphoprotein (with phosphorylation at the S26, T38, S101, S103, S107, and S109 sites) of PPP1R14A (NP_001230876.1) between primary tumor and normal tissues, respectively, by entering “PPP1R14A”. The PPP1R14A promoter methylation levels among different cancers and corresponding adjacent tissues were evaluated thereafter. The significant differences were determined using Student-test, with *p* <0.05 considered statistically significant. Datasets of six tumors were selected, namely, breast cancer, ovarian cancer, colon cancer, KIRC, UCEC, and LUAD for the analysis.

### Correlation Between Protein Phosphatase 1 Regulatory Inhibitor Subunit 14A Expression Profiles and Different Pathological Stages

In addition, violin plots of the PPP1R14A expression in different pathological stages (stage I, stage II, stage III, and stage IV) of all TCGA tumors *via* the “Stage Plot” module in “Expression DIY” of GEPIA2 (http://gepia2.cancer-pku.cn/#index) (Tang et al., 2019) were obtained. By entering “PPP1R14A” into the “Gene” input box and selecting the corresponding datasets into the “Datasets” box, the relationships between PPP1R14A and different pathological stages across cancers were evaluated. The log2 [TPM (Transcripts per million) +1] transformed expression data were applied for both the violin and corresponding box plots.

### Prognostic Analysis

The linkages between the PPP1R14A expression and the prognosis of patients, including overall survival (OS), disease-specific survival (DSS), and progression-free interval (PFI) in 33 types of cancers were examined using Kaplan–Meier curves (Liu et al., 2018). The data for relevant analysis were obtained from TCGA. The hazard ratios (HRs) and 95% confidence intervals were calculated using univariate COX regression survival analysis. Statistical analysis and visualization were employed by R survival (version: 3.2-10) and survminer (version: 0.4.9) statistical packages.

### Genetic Alteration Analysis

After logging into the cBioPortal web (https://www.cbioportal.org/) (Cerami et al., 2012; Gao et al., 2013), we chose cases in “TCGA Pan-Cancer Atlas Studies”, with both mutations and copy number alterations (CNAs), and for the “Quick select” option, “PPP1R14A” was selected to further query the genetic alterations and characteristics of PPP1R14A. The alteration frequency, mutations, and CNAs status results across all TCGA tumors were observed in the “OncoPrint” and “Cancer Types Summary” modules. Moreover, the “Comparison/Survival” module was employed to obtain the data and diagrams for Kaplan–Meier plots with log-rank analysis on the overall, disease-free, disease-specific, and progression-free survival differences for the TCGA cancer cases with or without PPP1R14A genetic alterations.

### Receiver Operating Characteristic Analysis

For finer resolution, the performance of a diagnostic test over a range of possible values of a predictor variable, ROC curves, which provides a useful tool in evaluating early stages of a new diagnostic test, and the area under ROC curves (AUC) were employed, using R pROC (version: 1.17.0.1) and ggplot2 (version: 3.3.3) packages (Mandrekar, 2010; Robin et al., 2011), with displays appearing only when AUC surpassed 0.9.

### Pan-Cancer Analysis of the Correlation of Protein Phosphatase 1 Regulatory Inhibitor Subunit 14A Expression With Immune Cells Infiltration

The data of 33 types of cancer and normal tissues in TCGA were downloaded from the Genomic Data Commons (GDC) data portal website (https://portal.gdc.cancer.gov/). Immuneeconv, an R software package that integrates the two latest algorithms, TIMER and xCell (Li et al., 2016; Aran et al., 2017; Sturm et al., 2019; Wang et al., 2019; Zeng et al., 2019; Frost et al., 2020; Izzi et al., 2020; Li et al., 2020; Sturm et al., 2020; Zhang et al., 2020), was used to generate and evaluate reliable immune scores. In the visualized plot, the horizontal axis represents different tumor tissues, vertical axis represents diverse types of immune lymphocytes, different colors represent correlation coefficients, and the stronger the correlation the more intense the color. The significance of the two groups of samples passed the Wilcox test.

### Relationship of Protein Phosphatase 1 Regulatory Inhibitor Subunit 14A Expression Between Tumor Mutational Burden/Microsatellite Instability With Immune Checkpoint Molecules

The mRNA-seq data, comprising tumor mutational burden (TMB), microsatellite instability (MSI) scores, and the expression levels of immune checkpoint–related genes and PPP1R14A in corresponding samples, were obtained from TCGA (Bonneville et al., 2017; Thorsson et al., 2018). Correlation analyses between the PPP1R14A expression and TMB/MSI, immune checkpoint molecules (ICMs) were performed using Spearman’s method. In R ggstatsplot and pheatmap, packages were applied for analyzing and visualizing data. The significance threshold in this study was set using p value < 0.05.

### Protein Phosphatase 1 Regulatory Inhibitor Subunit 14A-Related Gene Enrichment Analysis

Using the query of a single protein name (“PPP1R14A″) and organism (“*Homo sapiens*”), we first searched in the STRING website (https://string-db.org/) (Szklarczyk et al., 2019) and set the following main parameters: network type (“physical network”), minimum required interaction score ["custom value (0.235)"], active interaction sources (“Textmining, Experiments, and Databases "), and maximum number of interactors (“no more than 50 interactors” in the first shell). Finally, the available experimentally and putatively determined PPP1R14A-interacting proteins were obtained. The “Similar Gene Detection” module of GEPIA2 was applied to obtaining the top 100 PPP1R14A-correlated targeting genes based on the datasets of all TCGA tumors and normal tissues. An intersection analysis with a Venn diagram output was performed to compare the PPP1R14A-correlated and interacted genes. The “Gene_Corr” module of TIMER2.0 was used to supply the heatmap data of overlapped genes, which contains the partial correlation (cor) and p-values in the purity-adjusted Spearman’s rank correlation test. The R clusterProfiler (version: 3.14.3) and org.Hs.eg.db packages (Yu et al., 2012) were used to carry out and visualize Gene Ontology (GO) and Kyoto Encyclopedia of Genes and Genomes (KEGG) analyses. To control false-positive discovery rates, modified p-values and Benjamini/Hochberg algorithm were introduced. p <0.05 and false discovery rate (FDR) <0.05 were considered statistically significant.

## Results

### Protein Phosphatase 1 Regulatory Inhibitor Subunit 14A Expression Landscapes Across Cancers

**FIGURE 1:**
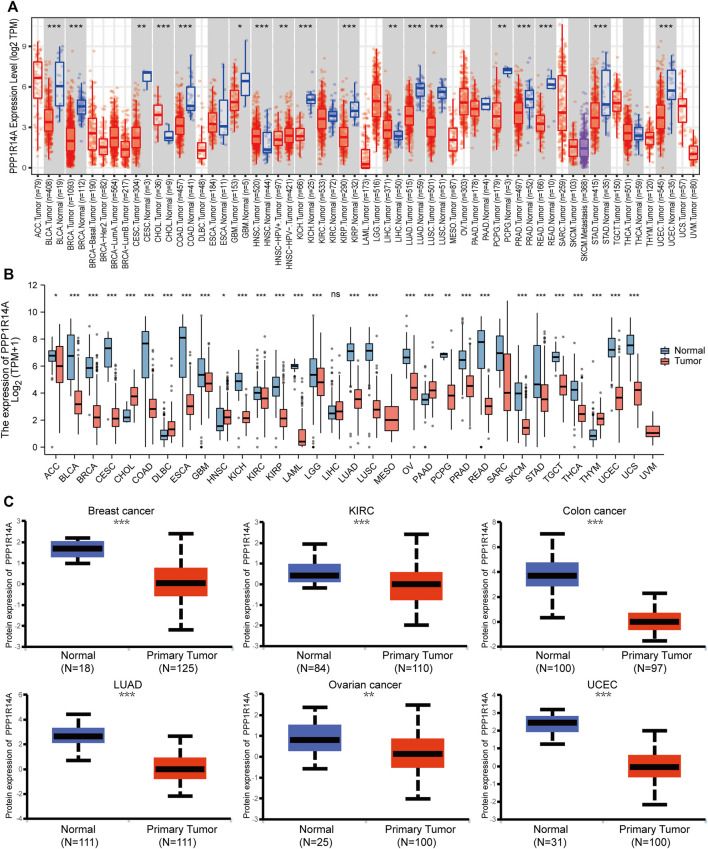
Aberrant mRNA expression of PPP1R14A in pan-cancer from the TIMER2.0 database indicated that the PPP1R14A expression in 18 cancer types. The red and blue boxes represent tumor tissues and normal tissues, respectively **(A)**. Expression level of PPP1R14A in different cancer types from TCGA and GTEx **(B)**. PPP1R14A protein expression level in normal tissues and primary tissues of breast cancer, KIRC, colon cancer, LUAD, ovarian cancer, and UCEC was examined using the CPTAC datasets **(C)**. ns, *p* ≥ 0.05; *, *p* < 0.05; **, *p* < 0.01; ***, *p* < 0.001.

**FIGURE 2:**
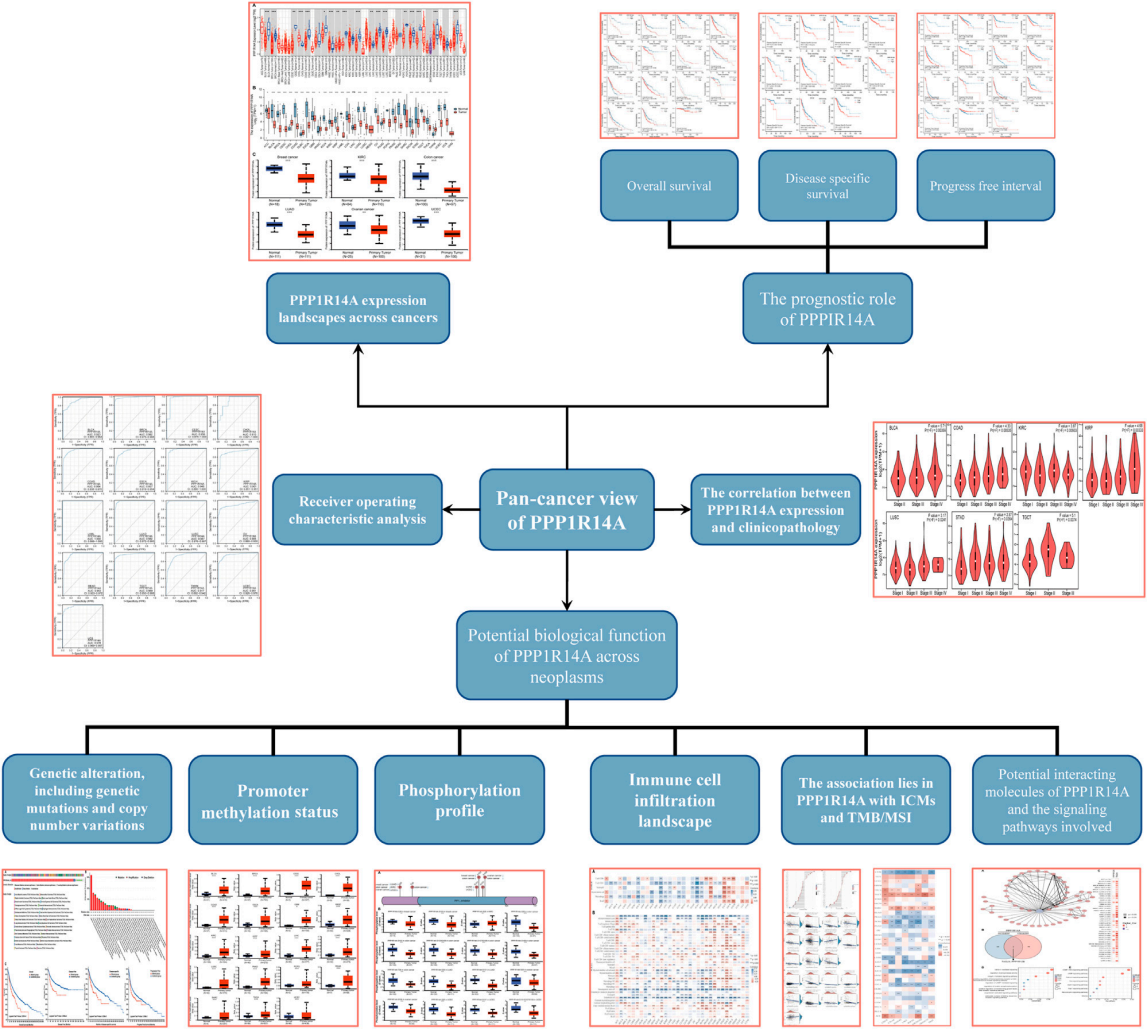
Schematic representation of the key findings in the study.

In this study, we found that PPP1R14A exhibited consistent mRNA expression in 23 different types of common human cancers, according to the results from the TIMER2.0 database. Significantly lower PPP1R14A expression in cancer versus adjacent normal tissues in BLCA, BRCA, COAD, KICH, KIRP, LUAD, LUSC, PRAD, READ, STAD, UCEC (*p* < 0.001), CESC, PCPG (*p* < 0.01), and GBM (*p* < 0.05) and higher in CHOL, HNSC (*p* < 0.001), and LIHC (*p* < 0.01) datasets were obtained (Figure 1A). We also compared the PPP1R14A expression using the data from the TCGA and GTEx databases. Downregulated PPP1R14A mRNA expression was observed in tumor tissues versus normal tissues in ACC, BLCA, BRCA, CESC, COAD, ESCA, GBM, HNSC, KICH, KIRC, KIRP, LAML, LGG, LUAD, LUSC, OV, PCPG, PRAD, READ, SKCM, STAD, TGCT, THCA, USEC, and UCS datasets, and the upregulated PPP1R14A expression profile was detected in CHOL, DLBC, HNSC, PAAD, and THYM datasets (Figure 1B). Further comparison of the PPP1R14A protein expression according to the CPTAC database demonstrated that the PPP1R14A protein expression was significantly decreased in advanced tumor tissues versus normal tissues in breast cancer, KIRC, colon cancer, LUAD, ovarian cancer, and UCEC (Figure 1C), and the schematic representation of the key findings in this study is shown in Figure 2.

### Correlation Between Protein Phosphatase 1 Regulatory Inhibitor Subunit 14A Expression and Clinicopathology as well as the Prognosis in Pan-Cancer

**FIGURE 3:**
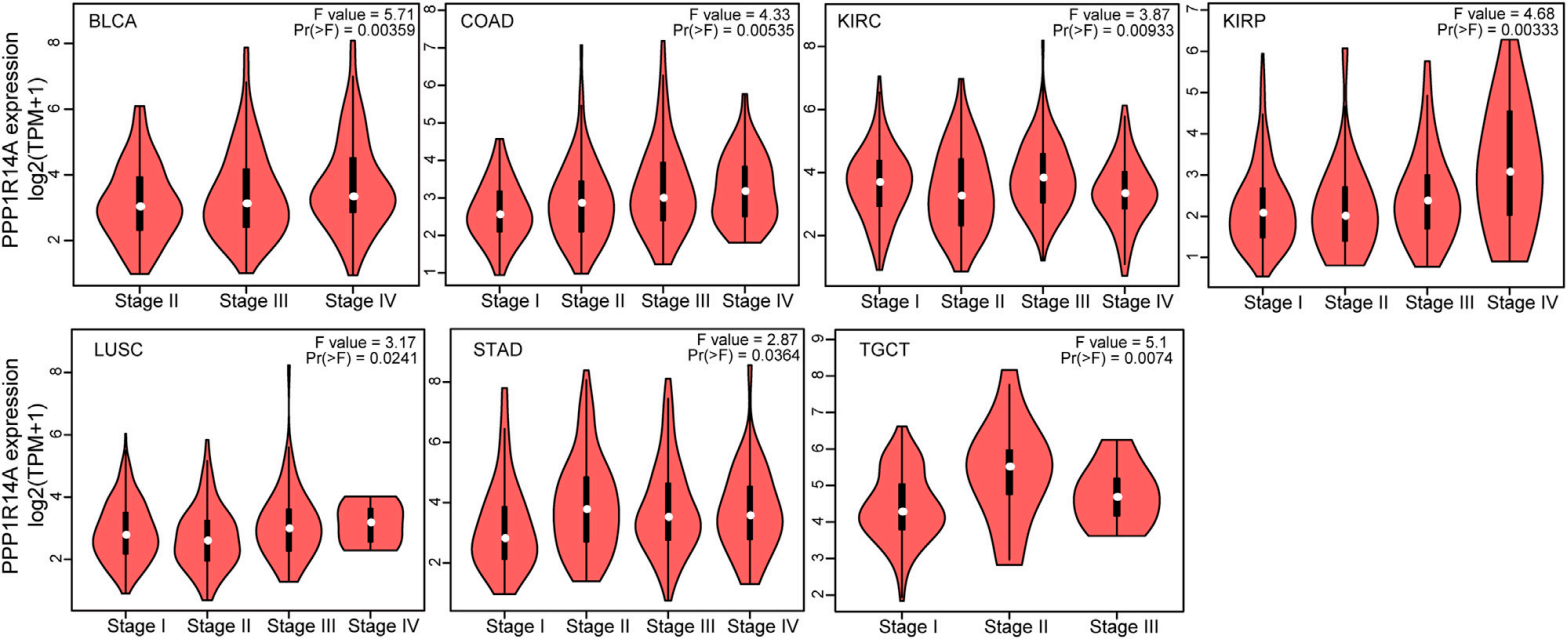
Correlations between the PPP1R14A expression and the main pathological stages, including stage I, stage II, stage III, and stage IV of BLCA, COAD, KIRC, KIRP, LUSC, STAD, and TGCT.

To investigate the association between the PPP1R14A expression and clinicopathological features in multiple cancers, we assessed the PPP1R14A expression in cancer patients of stages I, II, III, and IV. The TCGA database results revealed that the expression of PPP1R14A was significantly altered in BLCA (F = 5.71, *p* = 0.00359), COAD (F = 4.33, *p* = 0.00535), KIRC (F = 3.87, *p* = 0.00933), KIRP (F = 4.68, *p* = 0.00333), LUSC (F = 3.17, *p* = 0.0241), STAD (F = 2.87, *p* = 0.0364), and TGCT (F = 5.1, *p* = 0.0074) (Figure 3).

**FIGURE 4:**
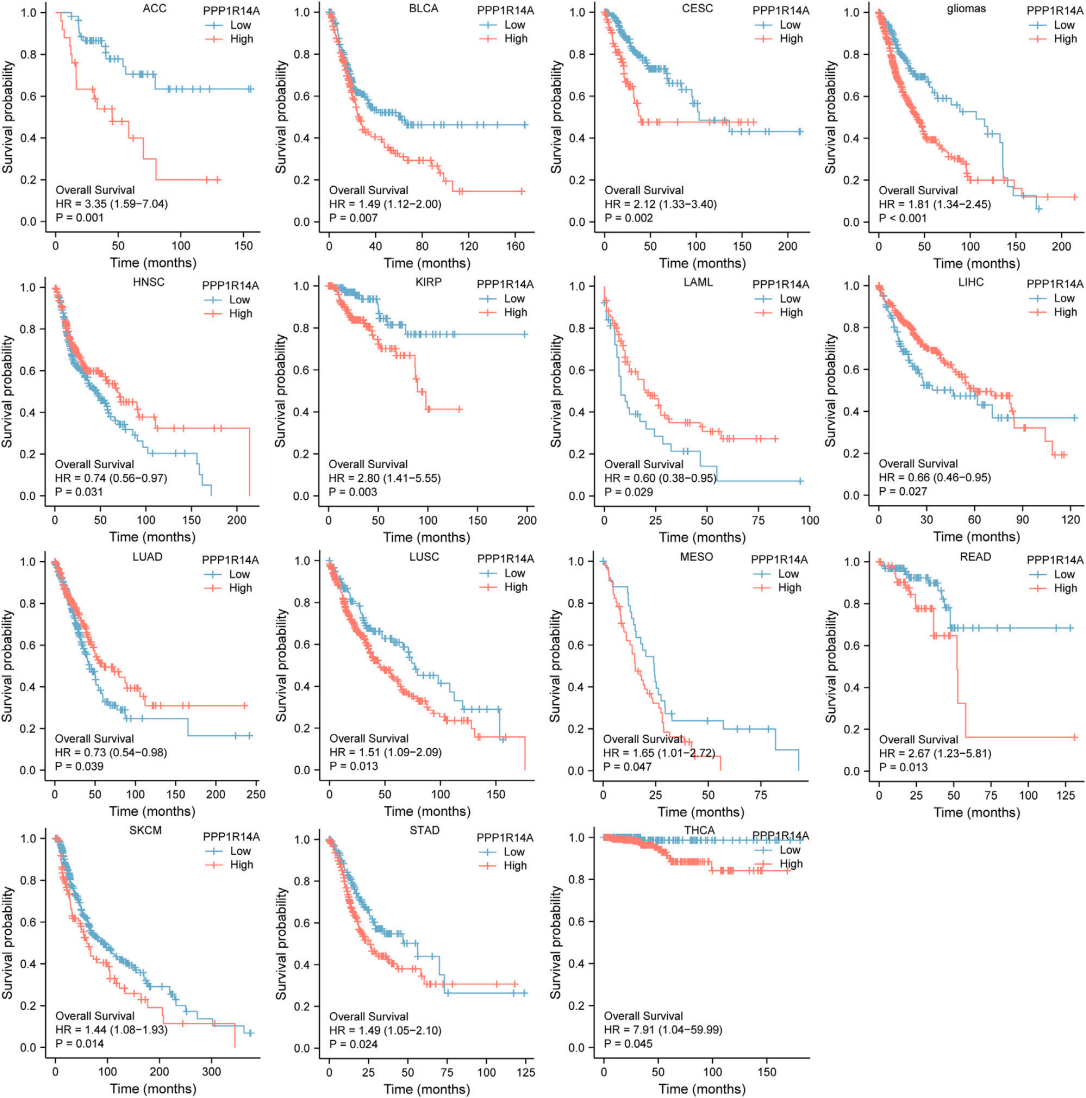
Association between the PPP1R14A expression and the OS of cancer patients. Kaplan–Meier survival curves for patients stratified by the different expressions of PPP1R14A in ACC, BLCA, CESC, gliomas, HNSC, LAML, LIHC, LUAD, LUSC, MESO, READ, SKCM, STAD, and THCA.

**FIGURE 5:**
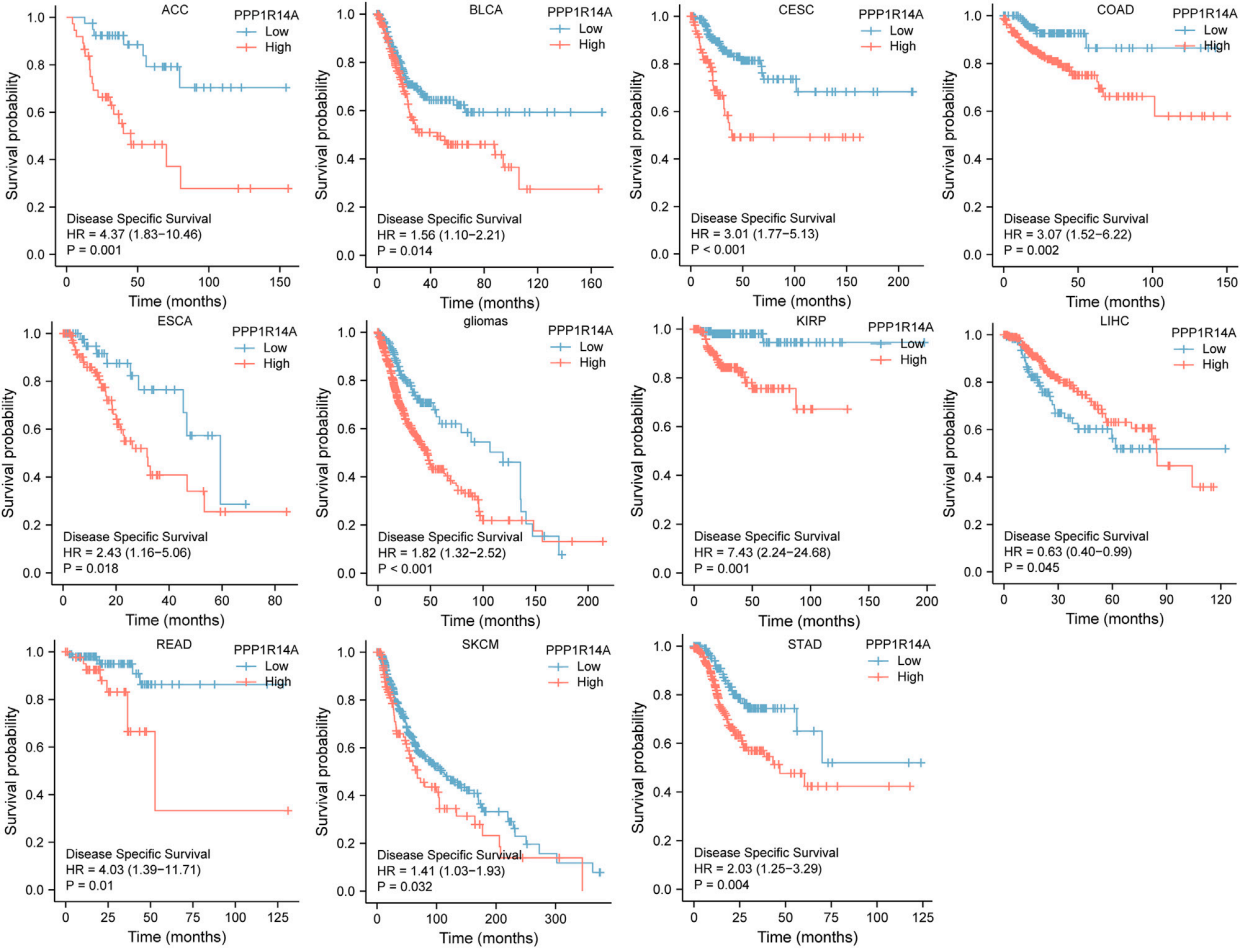
Association between the PPP1R14A expression and the DSS of cancer patients. Kaplan–Meier survival curves for patients stratified by the different expressions of PPP1R14A in ACC, BLCA, CESC, COAD, ESCA, gliomas, KIRP, LIHC, READ, SKCM, and STAD.

**FIGURE 6:**
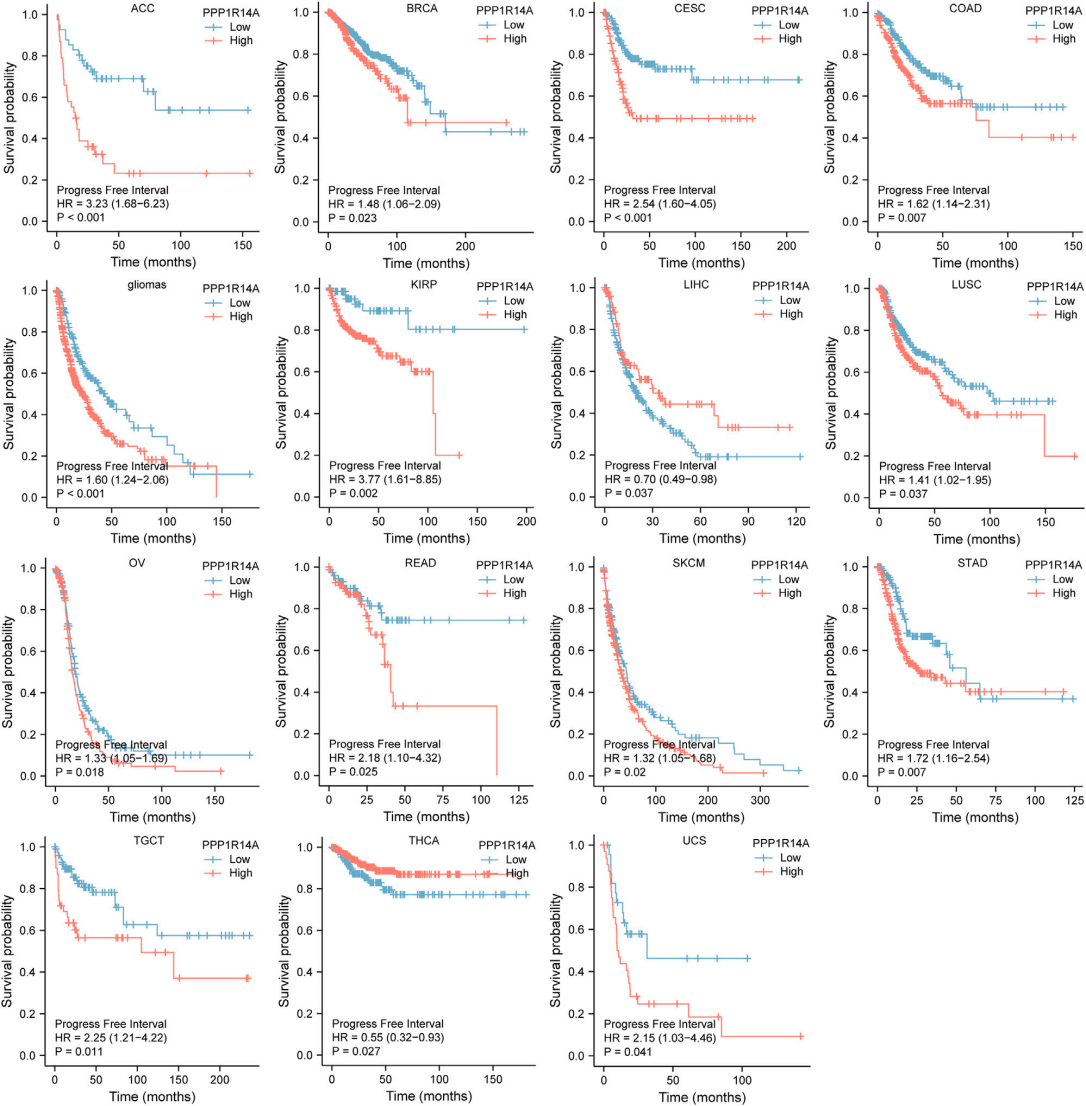
Association between the PPP1R14A expression and the PFI of cancer patients. Kaplan–Meier survival curves for patients stratified by the different expressions of PPP1R14A in ACC, BRCA, CESC, COAD, gliomas, KIRP, LIHC, LUSC, OV, READ, SKCM, STAD, TGCT, THCA, and UCS.

Next, we evaluated the relationship between PPP1R14A and the prognosis of patients across different cancers. Accordingly, PPP1R14A expression significantly acted as a risk factor for OS in ACC (*p* = 0.001), BLCA (*p* = 0.007), CESC (*p* = 0.002), gliomas (*p* < 0.0001), KIRP (*p* = 0.003), LUSC (*p* = 0.013), MESO (*p* = 0.047), READ (*p* = 0.013), SKCM (*p* = 0.014), STAD (*p* = 0.024), and THCA (*p* = 0.045) and protective factor in HNSC (*p* = 0.031), LAML (*p* = 0.029), LIHC (*p* = 0.027), and LUAD (*p* = 0.039) (Figure 4). Investigating the correlation of PPP1R14A expression with DSS of patients indicated that high expression of PPP1R14A unfavorably impacted DSS in ACC (*p* = 0.001), BLCA (*p* = 0.014), CESC (*p* < 0.0001), COAD (*p* = 0.002), ESCA(*p* = 0.018), gliomas (*p* < 0.001), KIRP (*p* = 0.001), READ (*p* = 0.01), SKCM (*p* = 0.032), STAD (*p* = 0.004), and friendly influenced LIHC (*p* = 0.045) (Figure 5). Analysis of the relationship between PPP1R14A expression and the PFI of patients across cancers was also conducted. Elevated PPP1R14A expression indicated poor outcomes for the PFI of patients in ACC (*p* < 0.001), BRCA (*p* = 0.023), CESC (*p* < 0.001), COAD (*p* = 0.007), gliomas (*p* < 0.001), KIRP (*p* = 0.002), LUSC (*p* = 0.037), OV (*p* = 0.018), READ (*p* = 0.025), SKCM (*p* = 0.02), STAD (*p* = 0.007), TGCT (*p* = 0.011), and UCS (*p* = 0.041) and signaled a positive prognosis in LIHC (*p* = 0.037) and THCA (*p* = 0.027) (Figure 6). In summary, these results show that PPP1R14A expression is significantly correlated with the patient disease prognosis.

### Receiver Operating Characteristic Analysis

**FIGURE 7:**
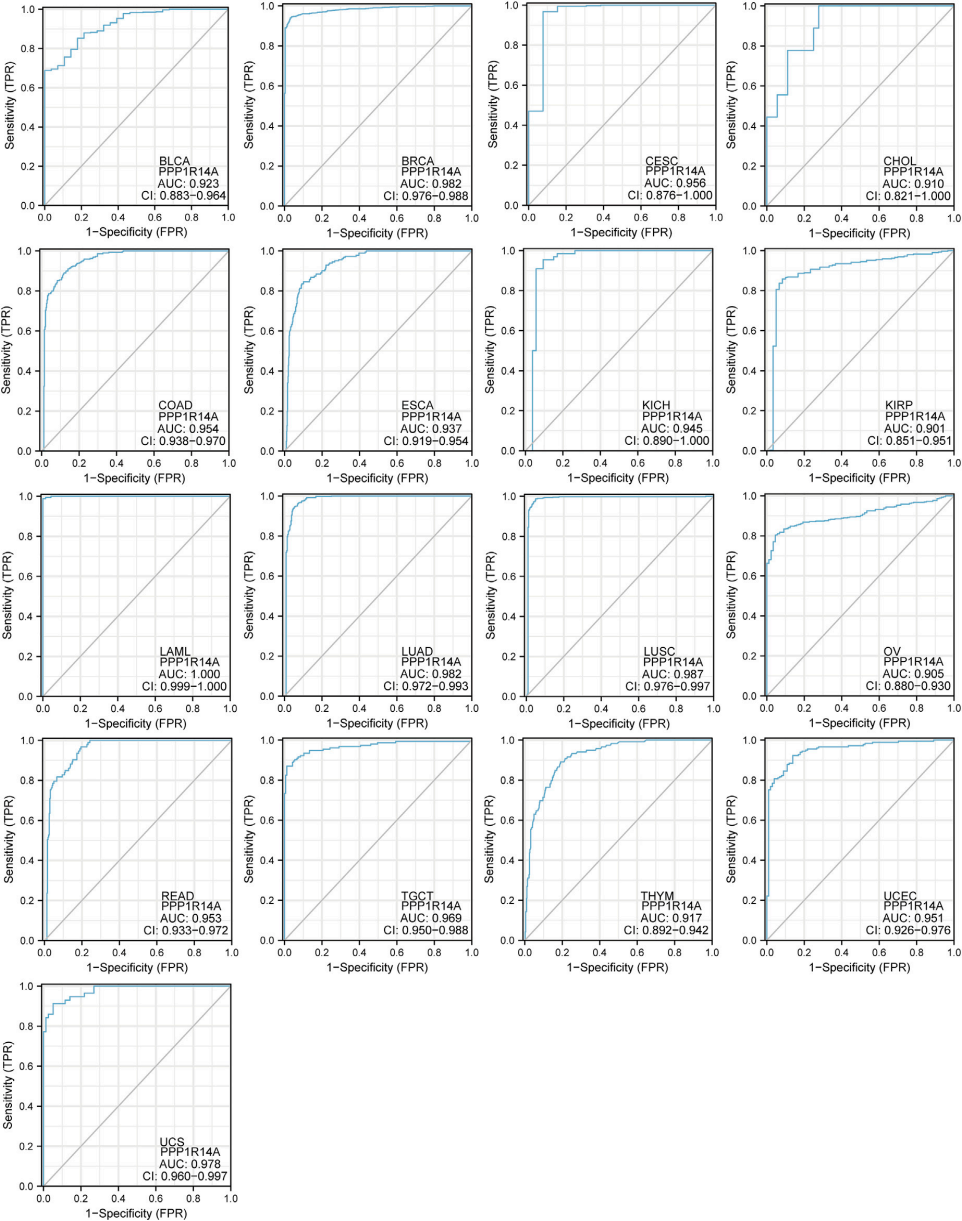
Receiver operating characteristic (ROC) curves for PPP1R14A across BLCA, BRCA, CESC, CHOL, COAD, ESCA, KICH, KIRP, LAML, LUAD, LUSC, OV, READ, TGCT, THYM, UCEC, and UCS.

To better understand the efficiencies of PPP1R14A prognostic prediction, we evaluated ROC curve analysis (Figure 7). Results demonstrated that BLCA (AUC = 0.923), BRCA (AUC = 0.982), CESC (AUC = 0.956), CHOL (AUC = 0.910), COAD (AUC = 0.954), ESCA (AUC = 0.937), KICH (AUC = 0.945), KIRP (AUC = 0.901), LAML (AUC = 1.000), LUAD (AUC = 0.982), LUSC (AUC = 0.987), OV (AUC = 0.905), READ (AUC = 0.953), TGCT (AUC = 0.969), THYM (AUC = 0.917), UCEC (AUC = 0.951), and UCS (AUC = 0.987), indicating that PPP1R14A may play an important role in the diagnosis and prognosis of these tumors.

### Genetic Alteration, Methylation Status, and Phosphorylation Profile of Protein Phosphatase 1 Regulatory Inhibitor Subunit 14A Across Multiple Tumors

**FIGURE 8:**
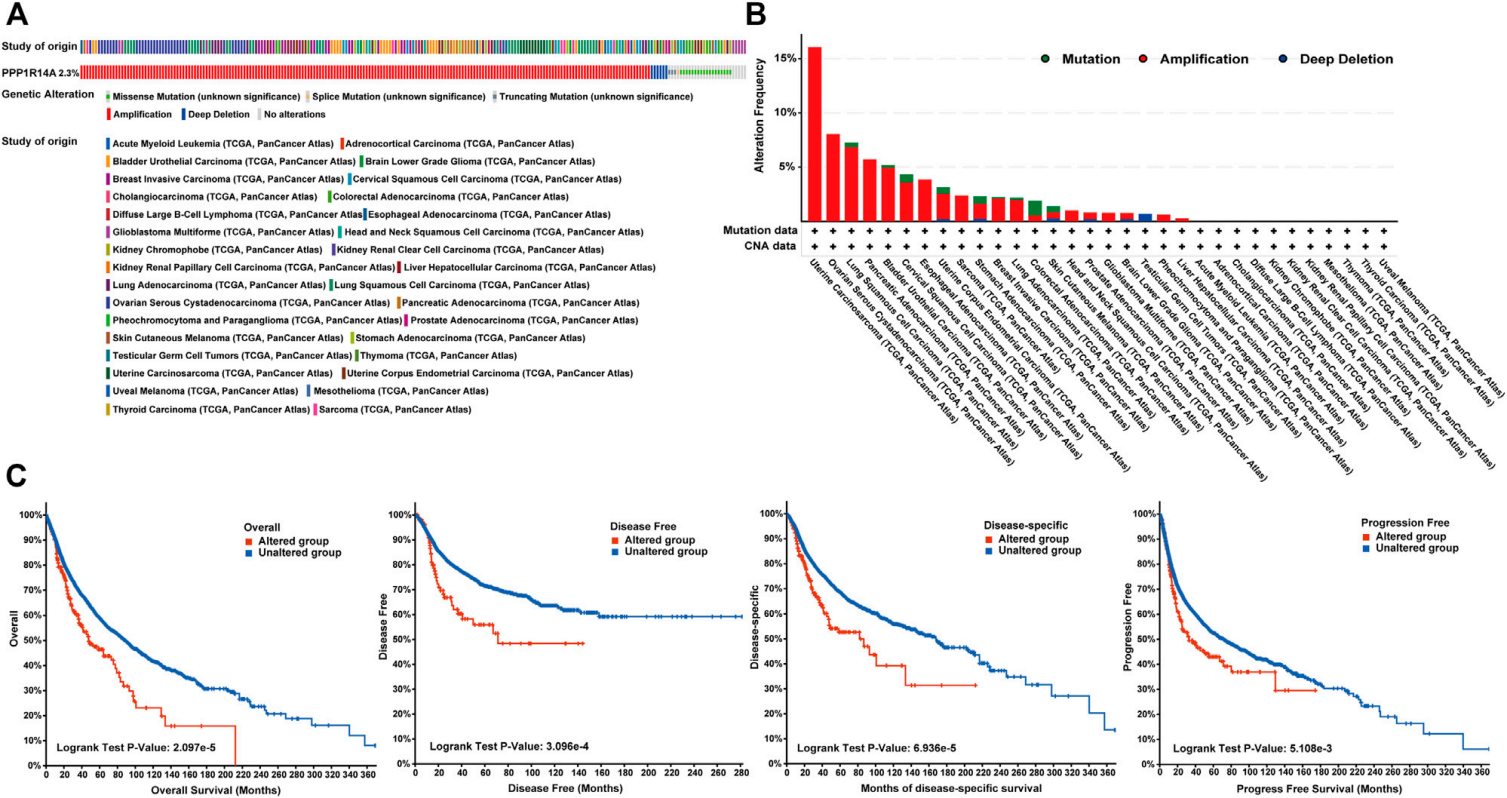
Summary of genetic alterations of PPP1R14A in different tumors. **(A)** Alteration frequency with different types of copy number variations and mutations examined **(B)**. Effect of PPP1R14A alteration status on overall, disease-free, disease-specific and progression-free survival of cancer patients investigated **(C)**.

**FIGURE 9:**
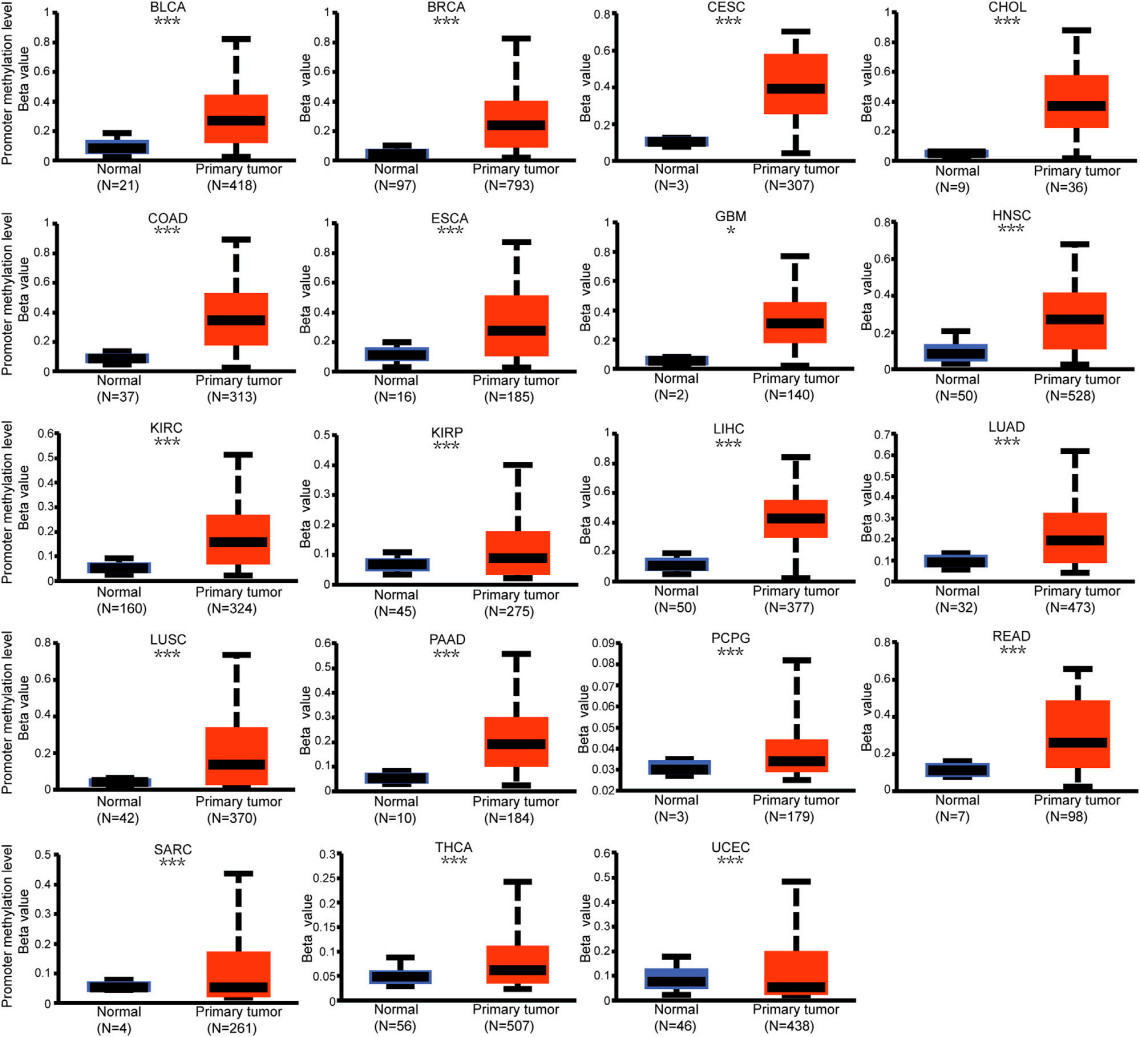
Promoter methylation level of PPP1R14A in pan-cancer. The results were obtained from the UALCAN database. ∗, *p* < 0.05; ∗∗, *p* < 0.01; ∗∗∗; *p* < 0.001.

Genetic alteration and methylation status were closely related to the initiation and progression, which affect the prognosis of tumors. In current study, we investigated the pan-cancer alterations of PPP1R14A using the cBioPortal (TCGA, Pan-Cancer Atlas) database and demonstrated that the alteration frequency of PPP1R14A was 2.3% across 32 various cancers and was prominent, 16.07% in samples of uterine carcinosarcoma. (Figures 8A,B). Among the different types of genetic alterations, amplification was the most common type detected. The potential relationship between genetic alterations in PPP1R14A and the prognosis of patients with different types of cancer was investigated (Figure 8C), revealing that tumor patients with genetic alterations in PPP1R14A had worse OS, DFS, DSS, and PFS than patients without alterations. Using the UALCAN database, we also investigated the promoter DNA methylation of PPP1R14A. A significant increase in the methylation levels within promoter regions of PPP1R14A was observed in BLCA, BRCA, CESC, CHOL, COAD, ESCA, GBM, HNSC, KIRC, KIRP, LIHC, LUAD, LUSC, PAAD, PCPG, READ, and THCA tissues compared to normal tissues. PPP1R14A promoter methylation levels in SARC and UCEC decreased (Figure 9).

**FIGURE 10:**
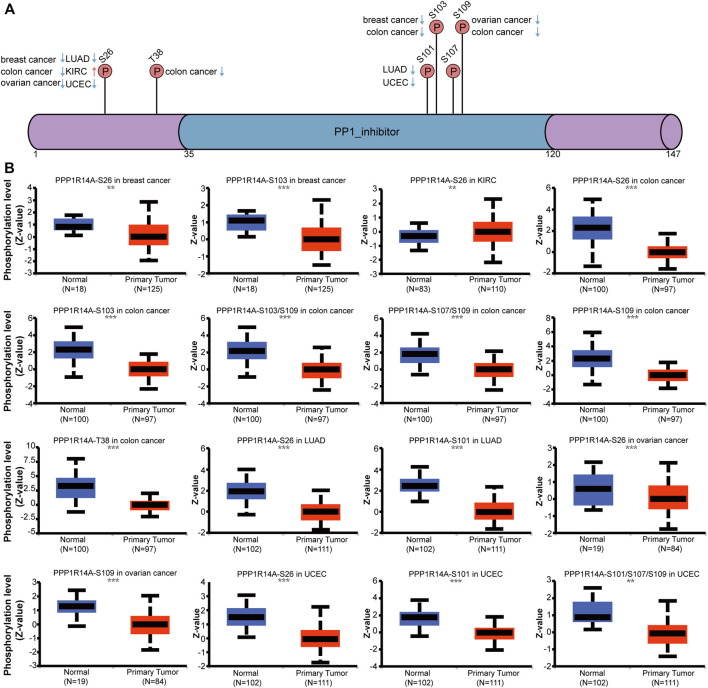
Phosphorylation of PPP1R14A in several selected cancers according to the CPTAC database. The schematic diagram and phosphorylation sites of the PPP1R14A protein are shown **(A)**. Phosphorylation of PPP1R14A at S26, S101, S101/S107/S109, S103, S103/S109 S107/S109, S109, and T38 was analyzed in breast cancer, clear cell RCC, colon cancer, LUAD, ovarian cancer, and UCEC **(B)**. ∗∗, *p* < 0.01; ∗∗∗, *p* < 0.001.

Phosphorylation modification levels regulating cancers progression and PPP1R14A exist various phosphorylation sites. In our study, we examined alterations in PPP1R14A phosphorylation levels between primary tumor tissues and normal tissues (Figure 10) with the CPTAC database, which includes six types of cancer, namely, breast cancer, KIRC, colon cancer, LUAD, ovarian cancer, and UCEC. Lower levels of S26 phosphorylation of PPP1R14A were observed in breast cancer, colon cancer, LUAD, ovarian cancer, and UCEC samples compared to normal samples. In contrast, S26 phosphorylation of PPP1R14A was increased in KIRC. T38 phosphorylation of PPP1R14A was decreased in colon cancer tissues compared to normal tissues. S101 phosphorylation of PPP1R14A was remarkably decreased in LUAD and USEC compared to normal adjacent tissues. In addition, S103 phosphorylation of PPP1R14A was significantly decreased in breast cancer and colon cancer compared to normal adjacent tissues. Moreover, S109 phosphorylation of PPP1R14A was remarkably decreased in ovarian cancer and colon cancer compared to normal adjacent tissues. Decreased S103 and S109 phosphorylation as well as S107 and S109 phosphorylation in PPP1R14A were observed in colon cancer, respectively. S101, S107, and S307 phosphorylation of PPP1R14A were significantly increased in USEC. These findings suggest that the phosphorylation of the S26, T38, S101, S103, S107, and S109 residues of PPP1R14A plays a crucial role in oncogenesis.

### Immune Cell Infiltration Landscape and Pan-Cancer Analysis of the Association Lies in Protein Phosphatase 1 Regulatory Inhibitor Subunit 14A With Immune Checkpoint Molecules and Tumor Mutational Burden/Microsatellite Instability

**FIGURE 11:**
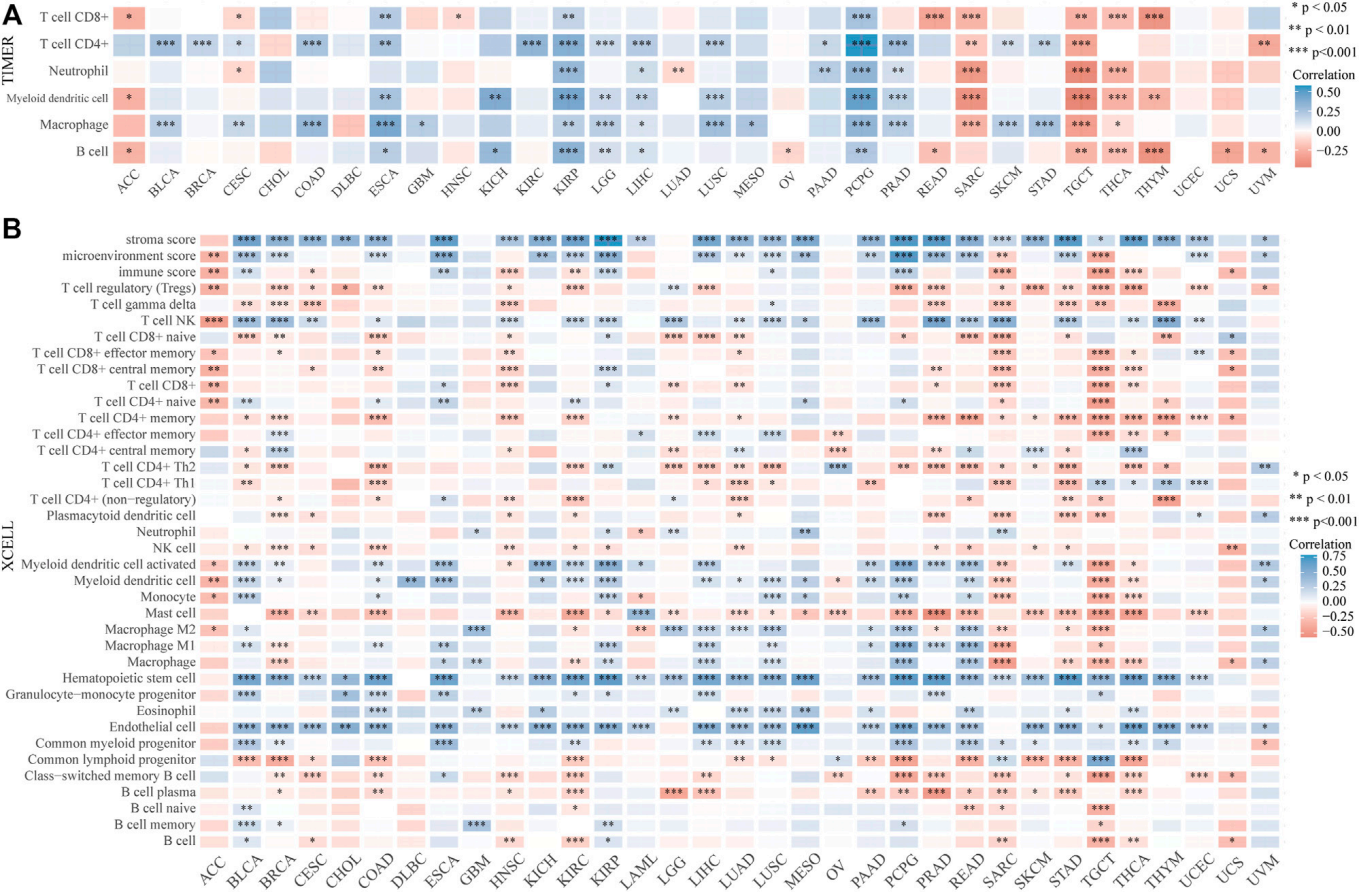
PPP1R14A expression correlated with immune infiltration. The PPP1R14A expression significantly correlated with the infiltration levels of various immune cells in different tumors based on TIMER **(A)** and XCELL **(B)** algorithms.

**FIGURE 12:**
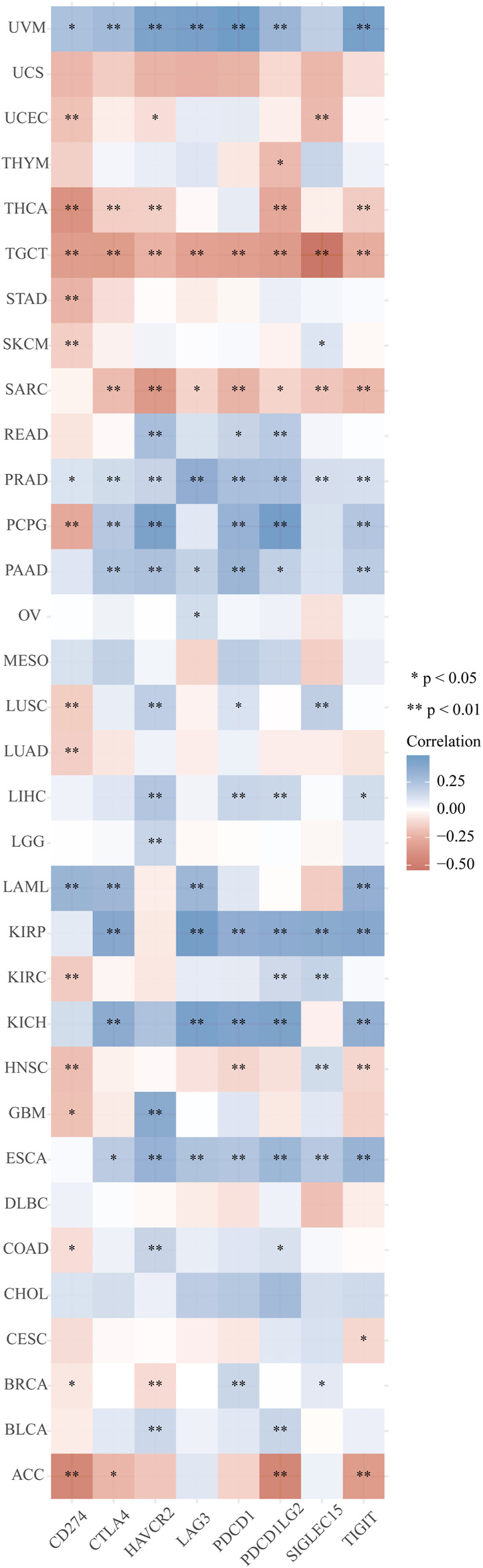
Correlation analyses of the PPP1R14A expression with immune checkpoint genes in pan-cancer.

**FIGURE 13:**
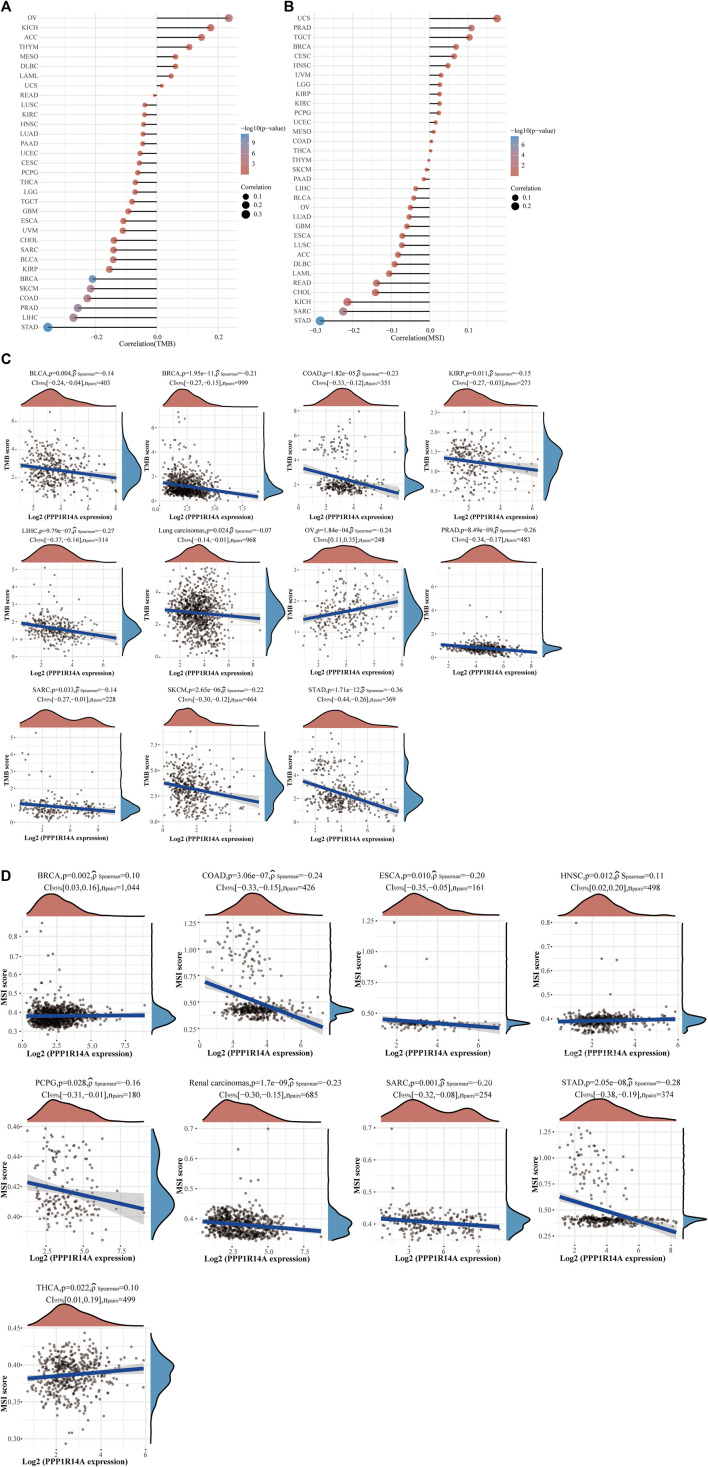
Correlation between the PPP1R14A gene expression and TMB and MSI in pan-cancer. A stick chart shows the relationship between the PPP1R14A gene expression and TMB in diverse tumors. The red curve represents the correlation coefficient, and the blue value represents the range **(A)**. A stick chart shows the association between the PPP1R14A gene expression and MSI in diverse tumors **(B)**. Relationship between the PPP1R14A gene expression and TMB **(C)** or MSI **(D)** in pan-cancer.

The distinct relationship between PPP1R14A and the immune response gave opportunity to perform a pan-cancer analysis of the association between PPP1R14A expression and the immune infiltration levels based on TIMER and XCELL algorithms. As shown in Figure 11A, the expression of PPP1R14A was significantly associated with the abundance of infiltrating immune cells, specifically, B cells in 14 types of cancer, CD4 + T cells in 18 types of cancer, CD8^+^ T cells in 11 types of cancer, macrophages in 17 types of cancer, neutrophils in 10 types of cancer, and DCs in 13 types of cancer. For further discrimination, using the xCell online tool to examine the relationship between PPP1R14A expression and the infiltration of different types of immune cell subtypes. Among 38 subtypes of immune cells, we found that the PPP1R14A expression negatively correlated with the following subtypes in SARC and TGCT positively and significantly associating with ESCA, KIRP, etc. In addition, hematopoietic stem cells, endothelial cells, and others were positively and T CD4^+^ memory cells and mast cells were negatively associated with the PPP1R14A expression in these different cancers (Figure 11B). To estimate the relationship between the PPP1R14A expression and the tumor environment across carcinomas, further investigation of the relationships between the PPP1R14A expression and immune checkpoint molecules (ICMs) were conducted. The expression of PPP1R14A negatively correlated with most ICMs in SARC and TGCT. In contrast, the expression of PPP1R14A positively correlated with most ICMs in ESCA, KICH, KIRP, PRAD, and UVM (Figure 12). Tumor mutation burden (TMB) and microsatellite instability (MSI) are two emerging biomarkers associated with the immunotherapy responses. The relationship between the PPP1R14A expression and TMB was investigated. The expression level of PPP1R14A, in addition to the phenomenon of significant positive correlation in OV, negatively correlated with TMB in several tumors, including BLCA, BRCA, COAD, KIRP, LIHC, lung carcinomas, PRAD, SARC, SKCM, and STAD (Figures 13A,C). The correlation of the PPP1R14A expression with MSI was also investigated in 33 types of cancer, and it indicated that BRCA, HNSC, and THCA exhibited positive correlations, while COAD, ESCA, renal carcinomas, SARC, and STAD exhibited negative correlations (Figures 13B,D).

### Protein Phosphatase 1 Regulatory Inhibitor Subunit 14A-Related Gene Enrichment Analysis

**FIGURE 14:**
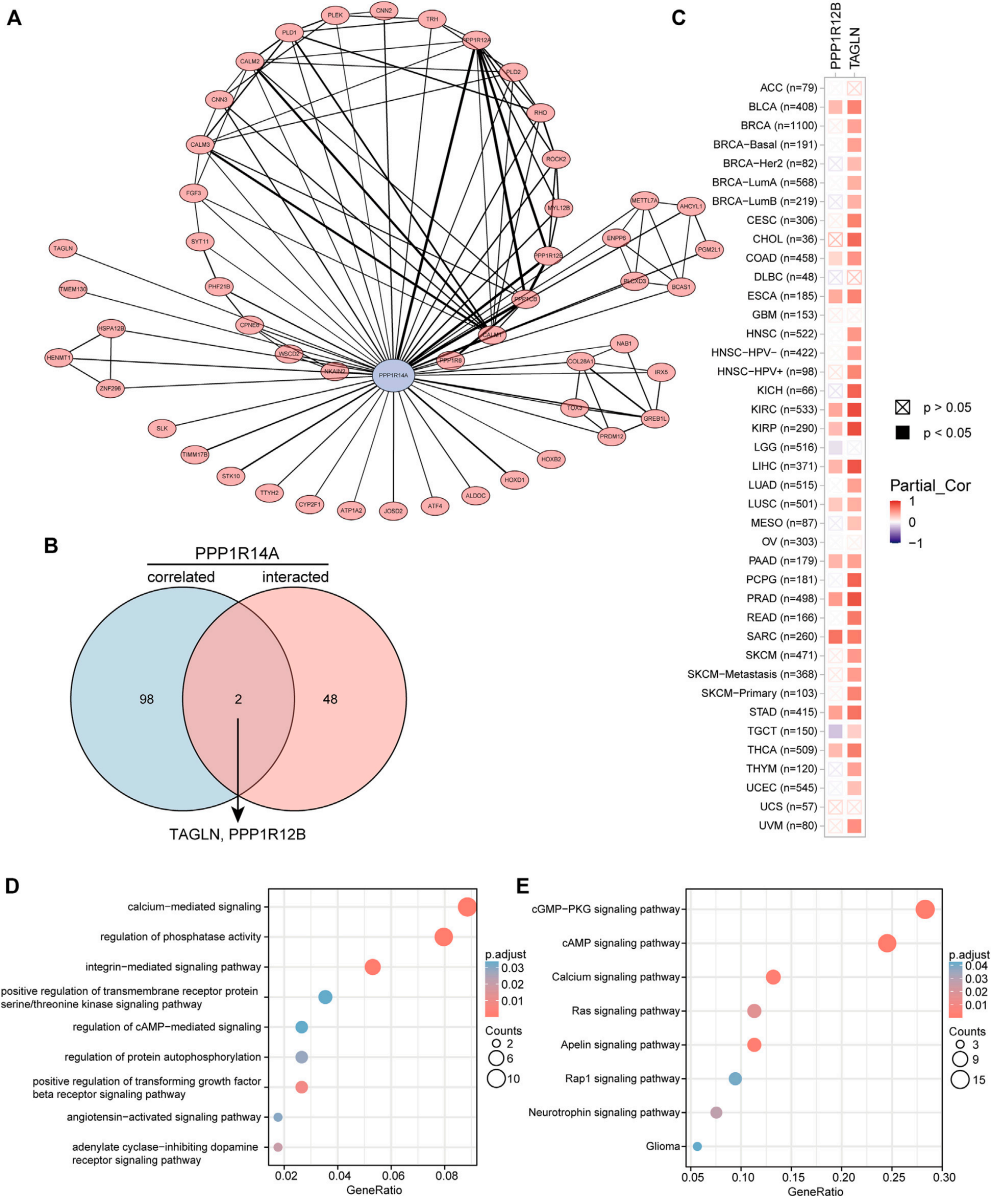
PPP1R14A-related gene enrichment analysis. A PPI network displays the available experimentally determined PPP1R14A-binding proteins. PPP1R14A is marked by blue color, line thickness represents combined score **(A)**. Using the GEPIA2 approach, we also obtained the top 100 PPP1R14A-correlated genes and constructed a Venn diagram with molecules that have a physical binding relationship with PPP1R14A, and it is found that there are two molecules, including TAGLN and PPP1R12B in the overlapping area **(B)**. Corresponding heatmap exhibits the relationship between PPP1R14A and the two intersecting molecules in the detailed cancer types **(C)**. Based on the PPP1R14A-binding and related genes, GO **(D)** and KEGG **(E)** pathway analysis was performed and displayed through bubble charts.

To better understand the interplay among the PPP1R14A-related genes, STRING database screening and PPI network with 119 edges and 51 nodes construction were performed, and visualization was carried out using Cytoscape (Figure 14A). Furthermore, the GEPIA2 approach was performed and we obtained the top 100 PPP1R14A-correlated genes, thereby constructing a Venn diagram with molecules that have a putative physical binding relationship with PPP1R14A, and discovered that there are two molecules, including TAGLN and PPP1R12B, in the overlapping area (Figure 14B). The corresponding heatmap exhibits the relationship between PPP1R14A and the two intersecting molecules in the various cancer types (Figure 14C).

Consistent with PPI network analysis, functional enrichment clustering of these genes showed a strong association with calcium-mediated signaling, regulation of phosphatase activity, and a variety of signaling pathways including, integrin-mediated signaling pathway, positive regulation of transforming growth factor beta receptor, adenylate cyclase-inhibiting dopamine receptor, regulation of protein autophosphorylation, angiotensin-activated signaling pathway, regulation of cAMP-mediated, positive regulation of transmembrane receptor protein serine/threonine kinase in GO as well as cGMP-PKG, cAMP, apelin, calcium, Ras, neurotrophin, Rap1, and glioma in KEGG (Figures 14D,E).

## Discussion

PPP1R14A, a widely expressed serine-threonine phosphatase regulating various cellular processes such as actin contraction, glycogen metabolism, cell cycle, protein synthesis, neuronal signal transduction, and many others, is also an inhibitor of protein phosphatase 1(PP1) (Eto, 2009; Virshup and Shenolikar, 2009). In recent years, emerging research have reported on the functional associations of PPP1R14A and clinical diseases, especially tumors (Riecken et al., 2016; Xu et al., 2020). However, the published research is limited to schwannoma, mesothelioma, and some malignancies of the gastrointestinal tract. Drawing on these findings, it is difficult to gain qualitative and quantitative understanding of the role of this molecule in other tumors. What specific role does PPP1R14A play in the pathogenesis of different tumors? Elucidation of the various molecular mechanisms or pathways remains unclear to date. To provide the research community with a macro level, broad overview of the molecular characteristics of PPP1R14A gene expression, survival prognosis, somatic mutations, CNAs, DNA methylation, and protein phosphorylation profile in various malignant tumors, a comprehensive bioinformatics exploration of PPP1R1AA in different tumors was performed.

Accordingly, an evaluation of the expression of this molecule at the mRNA and protein levels in various malignant tumors was examined. TIMER2.0, a database based on TCGA, is available with many tumor samples but lacks a control group. In conjunction with the GTEx database, a further study was launched, and the result showed that the expression of PPP1R14A was maladjusted in most tumors. It is worth noting that after combining more normal GTEx samples, the significantly high expression of PPP1R14A in LIHC disappeared. Whether this is a deviation caused by the normal samples of the analysis or a true objective observation needs to be further interrogated. In UALCAN, PPP1R14A showed a remarkably low protein expression in all six types of adult cancers tested, these results are consistent with the transcription level analyses.

The analysis of the prognostic survival of PPP1R14A in various cancers revealed a strange phenomenon, which was inconsistent with the general understanding of the molecule (Lu et al., 2021). For example, using Figures 4–6, we can observe that among BLCA, COAD, and KIRP patients, the PPP1R14A high expression group showed a worse prognosis. In contrast, in Figure 1, PPP1R14A in cancers of the same three categories, all showed a lower expression level, suggesting that this molecule may be a “tumor suppressor gene”. However, it exhibited a role in promoting cancer progression in patients with these three types of tumors, where upregulation of PPP1R14A promotes the malignant processes of tumors, thereby contributing to lower patient survival probabilities. This situation, surprisingly, is a microcosm. In most cases, the role of PPP1R14A when comparing tumor and normal samples is inconsistent with the role shown in the tumor cohorts, indicating that the role of PPP1R14A in these two processes, initiation and progression of cancers is opposite in most cases revealed when interpreting Figures 4–6. In Figure 3, a clear observation, confirming our viewpoints that as cancer progresses, the expression level of PPP1R14A gradually, and steadily increases in BLCA, COAD and KIRP. A counterexample is in KIRC. Overall, the expression trend of PPP1R14A in this tumor is ambiguous, meaning that it does not have the effect of promoting the progress of KIRC. The survival analysis of OS, DSS, and PFI were not significant. Overall, PPP1R14A may likely play completely different roles in the initiation and progression of most cancers, different from our general understanding of the molecule to date. Moreover, the ROC results indicate that PPP1R14A, whose AUC values surpass 0.9 in multiple malignant tumors such as BLCA, COAD, and KIRP, reached the outstanding level in the diagnostic test evaluation. Further, results with AUC greater than 0.7 were not displayed due to space limitations.

Since the genetic mutations and CNAs of somatic cells have been revealed to be closely related to the occurrence and development of tumors, there has been a trend to actively include molecules in cancer research globally. Our experiments indicate that PPP1R14A genetically alters in tumors. Although the frequency of genetic alterations is not as high as expected, it is enough to have a significant negatively influence on the prognosis of OS, DFS, DSS, and PFS. DNA methylation is a major form of epigenetic modification of DNA that regulates the gene expression without altering the sequence of DNA (Esteller, 2007; Toh et al., 2017; Wang et al., 2021). The link between DNA methylation of certain key genes and cancer has been gradually uncovered in the last two decades, including PPP1R14A, which has been experimentally demonstrated by Peng et al. As the expression of PPP1R14A in gastric cancer cell lines, are regulated by promoter region methylation (Peng et al., 2020). In addition, PPP1R14A was also evaluated for changes in the degree of methylation and the possibility of becoming tumor markers in other cancers, such as GBM, HNSC, and CRC (Bouras et al., 2019; Butler et al., 2020; Kanazawa et al., 2019; Li et al., 2017). These experiments indicated that PPP1R14A promoter methylation status was expected to become a prognostic and promising predictive biomarker. Meanwhile, our analysis, broadly explains the PPP1R14A promoter methylation level is significantly altered in BLCA, COAD, KIRP, and other cancers, giving clarity and expectation that the PPP1R14A could become a novel prognostic marker and potential therapeutic target. Hypermethylation within promoter regions often leads to the silencing or inactivation of tumor suppressor genes in cancerous cells (Esteller, 2007; Toh et al., 2017; Wang et al., 2021). Our results revealed that the methylation level of PPP1R14A is generally significant in most common cancers, whose profile is largely consistent with the downregulation of the expression levels of PPP1R14A as observed in Figure 1. However, we also noted many seemingly contradictory issues for further consideration. For example, in CHOL, HNSC and other malignant tumor samples, the methylation expression profile of the high promoter region of PPP1R14A is related to the high expression level of transcription. However, things in nature are often not that simple, and Smith et al. gave an overview of promoter DNA hypermethylation that guides gene expression activation, although it seems counterintuitive (Smith et al., 2020). Nevertheless, this may be a premature conclusion. The relationship between DNA methylation and PPP1R14A expression therefore requires further study.

Recent experiments have revealed that protein phosphorylation modification of genes often leads to pronounced changes in the function of the original protein molecules, inseparable from the origin and progression of malignant tumors (Im et al., 2021; Lin et al., 2021). Researches have reported that Rho-kinase (ROCK) participating in this phosphorylation process to a certain extent, CPI-17 Thr (38) phosphorylation can selectively inhibit myosin light chain phosphatase (MLCP), and then mediate the contraction process of smooth muscle (Eto, 2009; Satoh et al., 2011). Besides, studies have clarified that ROCK inhibitors showed a promising outlook on K-Ras–induced glioma, lung cancers and hematological malignancies (Nakabayashi and Shimizu, 2011; Rath and Olson, 2012). These results seemed to indicate that the phosphorylation process of PPP1R14A mediated by ROCK may be somewhat related to the occurrence and development of tumors. However, there is almost no research forthcoming to reveal the landscape of phosphorylated PPP1R14A in many cancers to date. On this basis, we analyzed it with UALCAN. The feedback results showed that in most cases, PPP1R14A tends to have low phosphorylation levels in tumors, suggesting that PPP1R14A phosphorylation may play a crucial role in tumorigenesis.

With in-depth exploration of the tumor immune microenvironment by scholars, one hidden immune checkpoint molecule after another is gradually emerging, which means that immunotherapy as a field of study is making progress. The advent of immunotherapy has transformed the clinical oncology landscape in recent years, with significant improvements in long-term survival in some patients. Despite this, the clinical application of immune checkpoint blockade in glioma patients remains challenging, mainly due to the “cold phenotype” of glioma and multiple factors inducing resistance (Qi et al., 2020). More suitable marker molecules therefore warrant urgently discovery and research. Among investigated biomarkers in immune checkpoint targeted therapy to date TMB and MSI have recently emerged as a promising predictor of response to immunotherapy in various tumor types (Snyder et al., 2014; Rizvi et al., 2015). Our results demonstrated that the expression pattern of PPP1R14A in cancers represented by ESCA, KIRP, and PCPG has a positive relationship with both ICMs and the infiltration of tumor immune lymphocytes. This indicate that although higher level of immune cell infiltration in tumors seems to be a good indicator, it seems to be “braked” by ICMs, and immunotherapy may be the key to releasing the brake pedal, and overcome the hurdle. Scrutinizing the relevant results in reverse needs to guide its significance for clinical treatment as well. The expression of PPP1R14A has a negative relationship with ICMs and immune cell infiltration levels in malignant tumors such as SARC or TCGT. It may, to some extent, indicate that the application of immunotherapy to the patients of these cancers with high PPP1R14A expression levels may not be effective. Yet, there is uncertainty, the next analysis reflected that PPP1R14A is negatively correlated with TMB/MSI in many tumors, which means that neoantigens, the target of immune cells, would be less in these cancer patients with high PPP1R14A, and immune checkpoint targeted therapy may be deflated as a result. The situation in our opinion is resolvable as we perceived that the degree of correlation is not as high as we anticipated. Coupled with the aid of clinical screening methods, procedure through immunotherapy can still be expected. Furthermore, it was worth noting that the results of TMB and MSI related to SARC gave warning that immunotherapy may not be really suitable for SARC patients.

To gain a deeper understanding of the molecular properties of PPP1R14A, we explored it and the molecules potentially related to it. The results of GO and KEGG indicated that these molecules are significantly enriched in many core pathways that lead to the initiation and progression of various malignant tumors, including calcium-mediated signaling, positive regulation of transforming growth factor beta receptor signaling pathway, cAMP signaling pathway, Ras signaling pathway, and so forth (Kadio et al., 2016; Batlle and Massagué, 2019; Shah et al., 2019). Have explained the role of PPP1R14A in tumorigenic transformation, which is basically achieved by inhibiting merlin activation, promoting Ras activation, these findings are consistent with our enrichment results Jin et al. (2006). Through the screening of the Venn diagram, we found two overlapping molecules, TAGLN and PPP1R12B with potential interaction with PPP1R14A. As shown in Figure 14C, PPP1R14A and TAGLN exhibit high and extensive synchronization in a variety of malignant tumors, especially renal carcinomas, implying that the two molecules are likely to work together, to indicate tumorigenesis. However, more explorations should be conducted to clarify the processes.

## Conclusion

Overall, this research has contributed to understanding the role of PPP1R14A in tumorigenesis from the perspective of clinical tumor samples. This initial pan-cancer analysis of PPP1R14A showed that PPP1R14A expression was statistically correlated with clinical prognosis, DNA methylation, protein phosphorylation, immune cell infiltration, and ICMs/TMB/MSI in multiple tumors and paves a way ahead for further research.

## Data Availability

Publicly available datasets were analyzed in this study. This data can be found here: https://portal.gdc.cancer.gov/.

## Glossary

ACC: Adrenocortical carcinoma
AUC: Area under ROC curves
BLCA: Bladder urothelial carcinoma
BRCA: Breast invasive carcinoma
CESC: Cervical squamous cell carcinoma and endocervical adenocarcinoma
CHOL: Cholangiocarcinoma
CNAs: Copy number alterations
COAD: Colon adenocarcinoma
CPI-17: 17 kDa PKC-potentiated inhibitory protein of PP1
CPTAC: Clinical Proteomic Tumor Analysis Consortium database
DLBC: B-cell lymphoma
DSS: Disease-specific survival
ESCA: Esophageal carcinoma
FDR: False discovery rate
GBM: Glioblastoma multiforme
GO: Gene Ontology
HNSC: Head and neck squamous cell carcinoma
ICMs: Immune checkpoint molecules
KEGG: Kyoto Encyclopedia of Genes and Genomes
KICH: Kidney chromophobe
KIRC: Kidney renal clear cell carcinoma
KIRP: Kidney renal papillary cell carcinoma
LAML: Acute myeloid leukemia
LGG: Brain lower grade glioma
LIHC: Liver hepatocellular carcinoma
LUAD: Lung adenocarcinoma
LUSC: Lung squamous cell carcinoma
MESO: Mesothelioma
MLCP: Myosin light chain phosphatase
MSI: Microsatellite instability
OS: Overall survival
OV: Ovarian serous cystadenocarcinoma
PAAD: Pancreatic adenocarcinoma
PCPG: Pheochromocytoma and paraganglioma
PFI: Progress-free interval
PPP1R14A: Protein phosphatase 1 regulatory inhibitor subunit 14A
PRAD: Prostate adenocarcinoma
READ: Rectum adenocarcinoma
ROC: Receiver operating characteristic
ROCK: Rho-kinase
SARC: Sarcoma
SKCM: Skin cutaneous melanoma
STAD: Stomach adenocarcinoma
TGCT: Testicular germ cell tumors
THCA: Thyroid carcinoma
THYM: Thymoma
TMB: Tumor mutational burden
UCEC: Uterine corpus endometrial carcinoma
UCS: Uterine carcinosarcoma
UVM: Uveal melanoma
